# Analyzing Impetus of Regenerative Cellular Therapeutics in Myocardial Infarction

**DOI:** 10.3390/jcm9051277

**Published:** 2020-04-28

**Authors:** Ming-Long Chang, Yu-Jui Chiu, Jian-Sing Li, Khoot-Peng Cheah, Hsiu-Hu Lin

**Affiliations:** 1 Department of Emergency Medicine, Taipei Medical University Hospital, Taipei 11031, Taiwan; shaulongking@gmail.com (M.-L.C.); cutejoin@hotmail.com (Y.-J.C.); 2 School of Medicine, College of Medicine, Taipei Medical University, Taipei 11031, Taiwan; 3 Department of Cardiology, Taipei Medical University Hospital, Taipei 11031, Taiwan; garuli1105@gmail.com

**Keywords:** myocardial infarction, stem cells, platelet-derived biomaterials, cardiac regeneration, cardiomyocytes

## Abstract

Both vasculature and myocardium in the heart are excessively damaged following myocardial infarction (MI), hence therapeutic strategies for treating MI hearts should concurrently aim for true cardiac repair by introducing new cardiomyocytes to replace lost or injured ones. Of them, mesenchymal stem cells (MSCs) have long been considered a promising candidate for cell-based therapy due to their unspecialized, proliferative differentiation potential to specific cell lineage and, most importantly, their capacity of secreting beneficial paracrine factors which further promote neovascularization, angiogenesis, and cell survival. As a consequence, the differentiated MSCs could multiply and replace the damaged tissues to and turn into tissue- or organ-specific cells with specialized functions. These cells are also known to release potent anti-fibrotic factors including matrix metalloproteinases, which inhibit the proliferation of cardiac fibroblasts, thereby attenuating fibrosis. To achieve the highest possible therapeutic efficacy of stem cells, the other interventions, including hydrogels, electrical stimulations, or platelet-derived biomaterials, have been supplemented, which have resulted in a narrow to broad range of outcomes. Therefore, this article comprehensively analyzed the progress made in stem cells and combinatorial therapies to rescue infarcted myocardium.

## 1. Introduction

**Figure 1:**
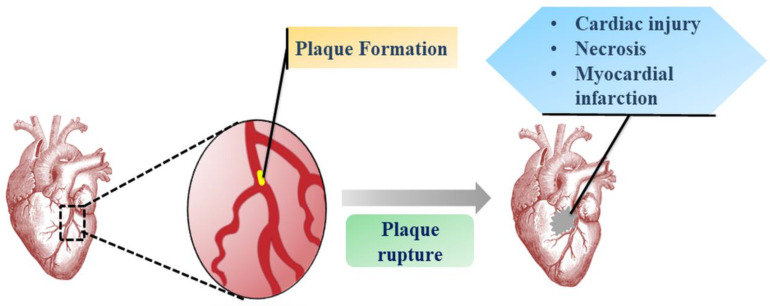
Pathogenesis of myocardial infarction (MI). The plaque formation in coronary artery reduces blood flow and after plaque rupture the developed blood clot leads to permanent damage of cardiomyocytes causing infarction.

Myocardial infarction (MI) is a central element of cardiovascular disorders with massive repercussions, such as left ventricular aneurysm, interventricular septal defect and acute mitral valve incompetence due to papillary muscle damage causing intractable heart failure (Figure 1) [1]. Though there is an endogenous cardiac repair system, the inability to regenerate injured or lost cardiomyocytes (CM) is an inherent biological limitation of the adult human heart that remains unaddressed by current pharmacotherapeutic advances. These strategies have only suppressed re-infarction rates and the underlying pathophysiology in terms of cardiac cell loss and undesired heart re-modelling has led to the recurrence of pertinent disorders [2,3]. Notwithstanding, heart transplantation seems to be a viable approach to limit the side effects of these treatment measures. However, it entails the limitation of donor availability, high cost, and other related risks of transplantation. 

**Figure 2:**
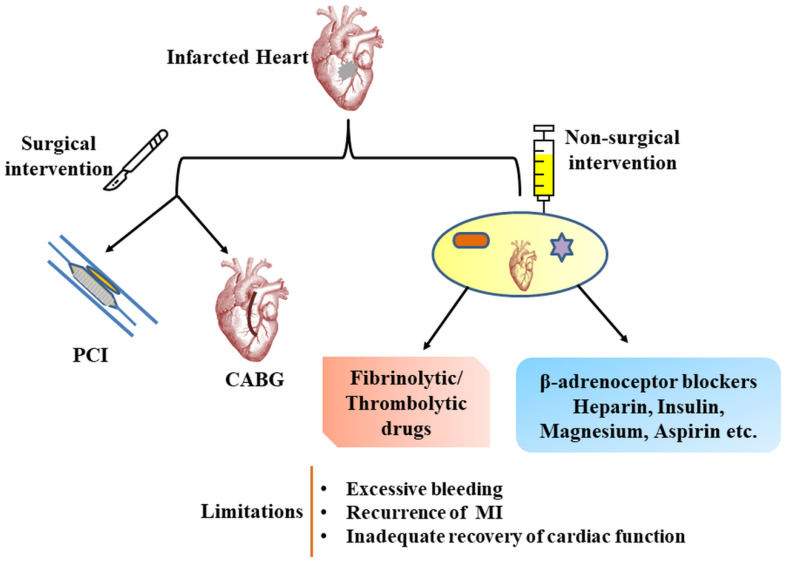
Conventional therapeutic surgical and non-surgical measures for addressing MI and their limitations. The selected approach relies on progression of MI and patient’s condition. CABG: Coronary artery bypass graft, PCI: Percutaneous coronary intervention.

To overcome the limitation of current therapeutic procedures (Figure 2) and to increase survival and efficacy rate in MI, novel regenerative therapeutic approaches are being explored using stem cells and platelet-derived biomaterials. Owing to their multi-differentiation potential, stem cells could be regulated and induced into CM for cardiovascular repair. Based on this fact, we have discussed below the various basic research and clinical studies on therapeutic potential of stem cells and their combination with stimulatory agents for regenerating CM to eliminate the risk of MI. 

## 2. Stem Cell-Based Anti-Myocardial Infarction Strategies

**Figure 3:**
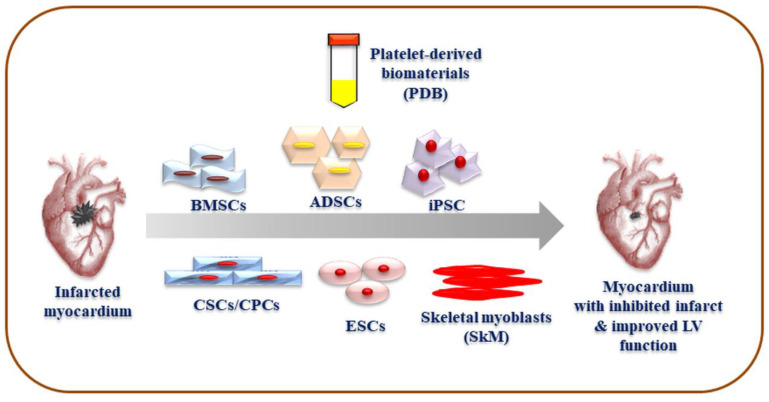
Stem cell and platelet-derived biomaterials-based bioengineering of infarcted myocardium. ADSCs: Adipose-derived stem cells, BMSCs: Bone marrow-derived stem cells, CPCs: Cardiac progenitor cells, CSCs: Cardiac stem cells, ESCs: Embryonic stem cells, iPSC: Induced pluripotent stem cells, LV: left ventricular, PDB: Platelet derived biomaterials, SkM: Skeletal myoblasts. These therapies aim to inhibit infarct regions and improve left ventricular function for successful cardiac recovery.

The choice of stem cells is specific to regeneration target, source availability, and technical potential to isolate and differentiate cells. So far, bone marrow (BM)-derived stem cells (BMSCs), adipose-derived stem cells (ADSCs), cardiac stem and progenitor cells, embryonic stem cells (ESCs), induced pluripotent stem cells (iPSCs), and skeletal myoblasts (SkM) have been extensively studied for their cardiogenic potential (Figure 3) [2,4,5]. It is highly desirable that stem cells should exhibit not only cardiac differentiation potential but also vasculogenic ability, both of which are imperative for cardiac repair [6]. Further, their therapeutic reproducibility and safety must be proven before establishing clinical practices. Notably, both the paracrine impacts of cellular growth factors (Table 1) and cell potential to differentiate into myocardial lineage and to integrate with innate cardiac cells seem to play a critical role in restoring heart functions [7].

A recent meta-analysis showed the potential of cell-based therapy in heart failure patients, in the terms of performance status and exercise capacity, left ventricular ejection fraction, and quality of life [20]. However; we have detailed below the major stem cell types and their efficacy through attenuating post-myocardial infarction remodeling.

### 2.1. Bone Marrow-Derived Stem Cells (BMSCs)-Based Repair of Infarcted Myocardium

**Figure 4:**
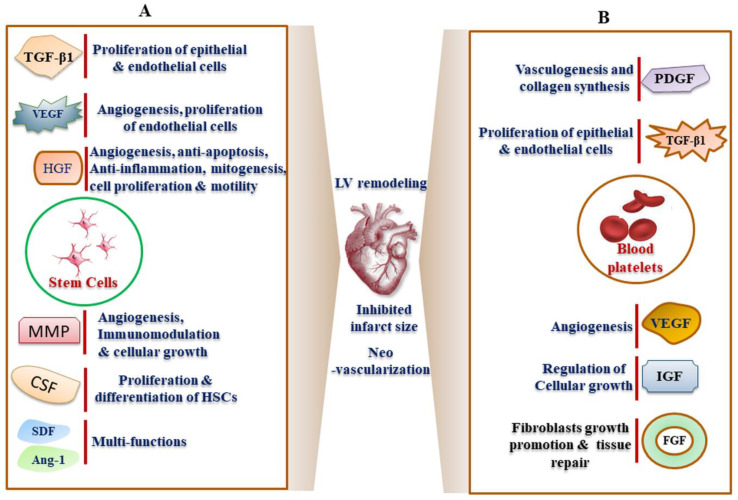
Cargo of growth factors releasate from (**A**) stem cells and (**B**) blood platelets which play a possible role in inhibiting myocardial infarct. The specific growth factors include angiopoietin (Ang-1), colony stimulating factor (CSF), fibroblast growth factors (FGF), insulin-like growth factors (IGF), hepatocyte growth factor (HGF), intercellular adhesion molecule (ICAM), matrix metalloprotease (MMP), platelet-derived growth factor (PDGF), stromal cell-derived factor 1 (SDF-1), transforming growth factor beta 1 (TGF-β1), vascular endothelial growth factor (VEGF), transforming growth factor (TGF) etc.

BMSCs seems to be possess a high potential in developing regenerative therapy for MI due to its abundance, safety and no ethical concerns regarding their use [5]. Moreover, protocols for the isolation, characterization, and maintenance of BMSCs are well established. Bone marrow cells are a mixed population of blood and stem cells, which are harvested through either direct aspiration or using cytokines such as granulocyte-colony stimulating factor factors during the peripheral mobilization of blood [5,21]. The first preclinical attempt to regenerate infarcted myocardium in mice was made in 2001, which proved to be a milestone in developing regenerative therapy for MI [22]. This study has been followed by various clinical and preclinical studies to develop safe, effective, and reproducible methods for MI treatment. A report demonstrated that BMSCs were able to differentiate myocytes and coronary arterioles in infarcted myocardium in mice [23]. Similarly, a review and meta-analysis of clinical studies also concluded that the myocardial injection of BMSCs following surgical revascularization might benefit patients with chronic ischemic heart disease with severely impaired left ventricular (LV) function [24]. The BMSCs have shown to be safe and with a comparable therapeutic efficacy in rabbit MI model when transplanted either via epicardial or intravenous routes [25], leading to their migration and differentiation into myocardial cells in the infarcted myocardium, and further inhibit subsequent vascular remodeling. Various discrepancies have been reported with respect to BMSCs doses and time gap between the onset of symptoms and initiation of MI treatment [26]. Nonetheless, studies have documented a maximum 10^7^ MSCs through percutaneous coronary intervention preferably within a week of MI to exploit therapeutic efficacy [27,28]. In addition to the short-term impact of BMSCs therapy, the recent clinical studies with long-term follow-up till 60 months have revealed significantly improved LV performance, ejection fraction, and quality of life [29]. This study reported decreased mortality with no any major side effects, and the patient’s ability to exercise also was significantly improved. A double-blind, placebo-controlled, and multicenter clinical study also reported that bone marrow derived progenitor not only inhibited the progression and relapse of MI, but also improved the contractility of infarcted left ventricular segments [30]. Generally, the transplanted BMSCs migrate, differentiate, and fuse to cardiomyocytes and endothelial cells, which further promote the development of a microenvironment through the release of paracrine signal such as vascular endothelial growth factor (VEGF), interleukin (IL)-6, platelet-derived growth factor (PDGF), fibroblast growth factor (FGF), hepatocyte growth factor (HGF), SDF-1, and insulin-like growth factor (IGF) (Figure 4A). These secretome also promotes homing and cardiac repair through the inhibition of inflammatory response, apoptosis, and oxidative stress [31,32,33,34]. Other well-known secretomes are extracellular, membrane-bound vesicles known as exosomes, which have been shown to synergize with stem cells and improved cardiac function, reduced infarct size, and increased neovascularization in acute MI [35]. An interesting study reported that low oxygen tension enhances the migration and homing ability of BMSCs to the injured region through activation of Kv2.1 channel and promotion of cell mobility focal adhesion kinase [36]. During MI recovery, BMSCs also inhibit activation of fibroblast, collagen deposition, extacellular matrix (ECM) and LV restructuring [37]. A seminal study showed that combined biotinylated and tethered IGF-1 and BMSCs significantly reduced cellular apoptosis and increased the expression of cardiac maturation protein in New Zealand rabbits MI model [38]. In a report, the myocardin-related transcription factor-A overexpressing BMSCs enhanced cardiomyocyte viability and reduced apoptosis induced by hydrogen peroxide exposure in rats [39,40]. Further, hydrogels have been employed to improve efficacy and safety of stem cells. BMSCs injected with hydrogel composite alpha-cyclodextrin/poly (ethyleneglycol)-b-polycaprolactone-(dodecanedioic acid)-polycaprolactone-poly (ethylene glycol) prevented LV remodeling and dilation, and improved local systolic and diastolic function in rabbit model of acute MI [41]. Studies on a porcine model also indicated that, compared to individual therapeutic successes, the synergistic effect of non-toxic alginate and myocardial extracellular matrix (ECM) may be an effective minimally invasive treatment alternative for MI [42]. Similarly, hyaluronic acid-based hydrogel also reduced LV remodeling and restored cardiac functions in ovine MI [43]. Furthermore, a recent meta-analysis of animal models with acute MI has documented the synergistic therapeutic intervention involving pharmaceutical and cell therapy using atorvastatin and BMSCs which resulted in increased capillary density of infarcted and peri-infarcted region through inhibited apoptosis, oxidative stress, and inflammation in the infarcted myocardium [44]. Though BMSCs-mediated regenerative therapy seems promising in MI treatment, and supports the safe and biodegradable profile of biomaterials in enhancing the impact of cell-based therapy, the challenges associated with the characterization of MSCs and their clinical translation still remains to provide benchmark [45]. However, a recent, randomized, and controlled phase II/III clinical trial reported that intramyocardial delivery of bone marrow-derived mononuclear cells and CD133+ cells during coronary artery bypass grafts in in patients with recent MI significantly improved LVEF and reduced systolic wall thickness without any risk [46]. This study provides initial proof of concept to design extensive clinical studies to validate the role of regenerative cells along with current therapeutic practices. In contrast, a randomized, multi-centric, and double blinded randomized clinical trial on ST-segment elevation myocardial infarction among MI patients indicated that bone marrow-derived mononuclear cells did not improve left ventricular remodeling and infarct size [47].

### 2.2. Adipose Tissue: the Cardiac Axis of Myocardial Regeneration 

Adipose derived stem cells (ADSCs) are multipotent and highly abundant stem cells which are derived from autologous adipose tissues through liposuction followed by enzymatic digestion and subsequent subculture [48]. In addition to comparable differentiation potential to BMSCs, the ADSCs could rapidly proliferate and differentiate into cardiomyocyte, endothelial cells, and cardiac-related lineages [49,50]. This cardiogenic ability might be attributed to the paracrine activities of its secretome such as VEGF, bFGF, IGF-1, Interferon-γ, and HGF [6,48,49]. The induction of ADSCs into cardiac cells have also been achieved by using cardiogenic agents such as 5-azacytidine (5-Aza), angiotensin II and TGF-β1, and transfection of ADSCs with T-box-18 gene [51,52,53]. Similarly, other reported agents like rosuvastatin, gherlin, exendin-4, S-nitroso-N-acetyl-d,l-penicillamine, NapFF-NO have also been employed [54,55,56,57]. Studies using rat models have implied that ADSCs could improve both the cardiac contractility as well as electrical stability [58], and the priming of ADSCs with 3,5-disubstituted isoxazoles, ISX1, exendin-4, and melatonin have improved myocyte differentiation and cardiac functions [59,60]. Additionally, hypoxia and inflammatory signals have shown to induce the ADSCs’s regenerative potential to cardiomyocyte via activation of JAK/STAT and MAPK signaling pathways [61]. Currently, three modes of stem cells transplantation, i.e., intramyocardial (IM), intravenous (IV), and intracoronary (IC), have been evidenced. However, the majority of studies have employed IV as IM delivery of ADSCs seems difficult. To overcome this limitation, hydrogels have been developed to assist IM-mediated delivery [48]. In contrast, one study has reported that, compared to human BMSCs, the ADSCs were more effective in recovering cardiac function in a Sprague Dawley (SD) rat model of MI. However, none of stem cells showed induced myocardial angiogenesis [62].

Other studies in animal model and human evaluated the therapeutic efficacy and safety of ADSCs in MI recovery [63,64]. Though most of the preclinical studies indicated its safety in reperfusion, infarct reduction, and functional recovery, glioma development has also been evidenced in mice model [65,66]. Collectively, ADSCs-based therapy seems more efficacious and affordable; however, challenges related to tumor formation, survival in micro-environment of infarcted regions and lack of broad spectrum clinical trials need to be addressed before establishing standard clinical regenerative procedures. 

### 2.3. Cardiac-Derived Stem Cells (CSCs)/ Cardiac Progenitor Cells (CPCs)-Based Regenerative Therapies in MI

Being isolated from cardiac tissues such as atrial appendages, epicardial adipose and endomyocardial tissues, CSCs seems more effective and safe in regenerating injured myocardial tissues architecture and function [67]. CSCs are c-kit+ stem cells and have the potential to differentiate into multiple cell lineages, such as smooth muscle cells, cardiomyocytes, and endothelial cells with angiogenesis [68]. The heart is not only the source of CSCs, but also of cardiac progenitor cells [69]. The CPCs have been shown to manifest a mixed immunophenotypic profile, such as stem cell antigen-1, Islet-1 (Isl-1), PDGF receptor-alpha (PDGFRα), GATA4, NKX2.5, Abcg2, cKIt+, FLK1, MEFK5, CD34, CD44, CD45, CD105 etc. [70]. The transplantation of CPCs may promote direct differentiation and proliferation of cardiac cells through its paracrine effect and cell fusion effect, in addition to the anti-apoptotic, immunomodulatory, and angiogenesis traits [21,71]. 

Owing to the regenerative potential of CSCs, attempts have been made to transplant human CSCs (hCSCs) in rat model of MI, which generated a chimeric heart containing myocardium consisting of myocytes, coronary resistance arterioles, and capillaries [72]. Whether the human heart exhibits a CSC pool that promotes regeneration after infarction was examined and concluded that it contains a CSC compartment, ischemic injury could activate CSCs. In the chronic ischemic cardiomyopathy, the loss of functionally competent CSCs might be attributed to progressive functional deterioration as well as the onset of terminal failure [73]. During allogeneic or xenogeneic cellular transplantation in a mice model, characteristics of CSCs such as low retention and engraftment of transplanted cells and the adverse effects of inflammation and immunoreaction were found to be improved by combining them with thermosensitive poly (N-isopropylacrylamine-co-acrylic acid) or P (NIPAM-AA) nanogel, which provide porous structure for various nutrients and oxygen and rescue stem cells from immune cells [74]. With regard to the cellular dose of CSCs, a concentration between 0.3 and 0.75 × 10^6^ has been demonstrated for the required efficacy in rat model [75]. Interestingly, no much improvement in LV function or structure was reported above this threshold and further increases in cellular dose seem harmful. To further improve the delivery, safety, and efficacy of CSCs, bio-materials like hydrogels seem to be one of potent tools. Hydrogels like poly (l-lactic acid) could be used as mat to synergistically deliver VEGF and CSCs for synchronized angiogenesis and cardiomyogenesis in cardiac recovery in MI model of SD rats [76]. To avoid the immunogenicity and/or tumorigenicity risks during cell transplantation, the encapsulated hCSCs in thermosensitive poly (N-isopropylacrylamine-co-acrylic acid) nanogel was employed in mouse and pig models of MI, and didn’t not elicit systemic inflammation or local T cell infiltrations and improved cardiac function via reducing scar sizes [77]. This evidence allows one to conclude that thermosensitive nanogels could carry stem cells and prevent them from immune cells attack. The isolated CSC-derived exosome from right atrial appendage of patients undergoing bypass surgery have revealed an increased capacity to stimulate endothelial tube formation in umbilical vein endothelial cells [78], which indicate its potential therapeutic role in MI. In a multicenter randomized, double-blind, and placebo-controlled clinical trial, it was reported that intracoronary infused allogeneic human CSC in patients with ST-segment elevation MI showed no significant advantage in reducing infarct size or indices of LV remodeling; however, without any safety issues [79]. The above-mentioned evidences imply that CSCs could reduce the risk of immunogenicity and therefore could be employed as a tool for regenerative therapy for MI. However, issues related to harvesting, delivery and clinical efficacy, safety, and practice need to be well established for the exhaustive exploration of its regenerative potential. 

### 2.4. Embryonic Stem Cells (ESCs) in MI Therapy

Human ESCs are pluripotent and have been induced to differentiate and proliferate in various cell lineages. This potential of ESCs has also been harnessed in cardiac recovery and LV remodeling. The embryonic inner cell biomass of embryo is a rich source of stem cells which have been proven to possess mesenchymal stem cells characteristics such as immunophenotypic profile. The potential of ESCs to differentiate into cardiomyocytes, endothelial and smooth muscle cells ad libitum render it a better choice for therapeutic purpose than other adult stem cells [80,81]. This was validated in a seminal preclinical study showing that the administered hESC-CMs after being grafted survived, proliferated, matured, aligned, and synthesized gap junctions with host cardiac tissue of infarcted rat and attenuated LV remodeling [82]. Similarly, murine cardiac-committed murine ESCs also grafted into infarcted myocardium of immunosuppressed and immunocompetent sheep, and differentiated into mature CM expressing connexins [83]. These outcomes of a large-animal model of MI further support the potential therapeutic use of ESCs in regenerating severely damaged myocardium. In a recent clinical case report, the human ESCs embedded in a fibrin patch were transplanted to the infarcted region and differentiated into cardiac cells in presence of bone morphogenetic protein-2 (BMP-2) and a fibroblast growth factor receptor inhibitor [84]. Moreover, ESCs-derived exosomes increased neovascularization, cardiomyocyte survival, and suppressed fibrosis in the infarcted mice heart [85]. An important report evidenced that both hESCs-derived cardiomyocytes and cardiovascular progenitors (hESC-CVPs) equally improved systolic function and ventricular dilation in rats without any large presence of human vessels in rat [86]. In macaque monkey, the administered hESC-CMs into the infarcted site of improved left ventricular function, while compared to human bone marrow-derived mononuclear cells (hBM-MNC), the most commonly employed clinical cell type, the efficacy of hESCs-CVPs was better [87]. These therapeutic efficacies of ESCs imply its futuristic potential of ESCs in regenerating tissues in the infarcted heart. However, their availability, ethical concerns, and standard procedure related issues still remains a challenge in developing ESCs-based regenerative therapy. 

### 2.5. Bioengineering Infarcted Myocardium Through Induced Pluripotent Stem Cells (iPSCs) 

To sidestep the destruction of human embryos, which is the major bottleneck to embryonic stem cell research, the induced pluripotent stem cells (iPSCs) have been introduced to gain widespread approval and support [88,89,90]. This unique cellular state transition from somatic cells to naive pluripotency is governed by an interconnected network of four transcription factors i.e. Oct4, Sox2, c-Myc and Klf4 [91]. Therefore, the intriguing idea of isolating abundant autologous adult stem cells from easily accessible sites and transforming them to pluripotent state has led to investigate its therapeutic regenerative efficacy towards restoring cardiac functions in infarcted heart. In a MI mouse model, the iPSCs-derived CPCs expressing fetal liver kinase-1 surface marker demonstrated favorable myocardial remodeling with more vascular structures and improved left ventricular function [92]. The iPSC-derived cardiomyocytes (iPSCs-CM) also showed cardiac protection in rat via attenuated myocardial remodeling with increased angiogenesis, reduced infarct size and inhibited fibrosis through metabolic paracrine activities [93]. Further, it has been found that electrical simulation could accelerate cardiac differentiation ability of human iPSCs through activation of Ca2+/PKC/ERK pathways leading to maturation of CM, which were functionally grafted with host cardiac tissues [94]. To further enhance therapeutic regenerative efficacies, polyethylene glycol hydrogel and erythropoietin were combined with iPSCs-CM which improved cardiac function in MI rat, and enhanced infarct thickness and muscle content even in the lack of donor-cell engraftment [95]. In a novel attempt, direct epicardial injection of hiPSC-CM spheroid and gelatin hydrogel led to retained and equally distributed hiPSC-CM in porcine myocardium [96]. A 3-D human heart muscle constructs from human hiPSC-CM and hiPSC-derived endothelial cells improved LV function by 31%, via CM proliferation, remuscularization, and possible electrical coupling in infarcted guinea pig myocardium [97]. The hiPSC-CM has also been shown to improve global as well regional recovery in cardiac function through improving oxygen consumption, myocardial bioenergetic vasculogenesis and reducing CM apoptosis in a porcine model of ischemic injury [98]. Furthermore, epicardially implanted fibroblast-derived hiPSCs electrically enhanced conduction suppressed LV-end diastolic pressure, increased anterior wall thickness in diastole, and improved indices of diastolic function in rats with chronic heart failure [99]. Interestingly, the human iPSCs-derived cardiac muscle patches in fibrin scaffold with tri-lineage cardiac cells significantly lessened infarct size and improved cardiac function in the swine MI, which were pertinent to reduction in left ventricular wall stress; however, this therapeutic intervention showed no significant changes in arrhythmogenicity [100]. Although these evidences imply that human iPSCs possess the potential in the sphere of stem cell-based regenerative medicine, more exhaustive pre-clinical and clinical studies are needed to establish its role in treatment of MI.

### 2.6. Cardiac Repairing Throughskeletal MYOBLASTS (SkM)

Cell-based clinical therapies have now opened the path to explore somatic cells such as skeletal myoblasts for their potential in cardiac recovery from MI [101]. SkM, being autologous, resistant to ischemic stress, and conducive to in vitro culturing, provides an opportunity to establish its role in cardiac remodeling. These are intermediate structure between basal lamina and sarcolema and normally present in quiescent phase in the heart [102]. Any trauma to heart muscle triggers its regenerative potential to restore functional and structural integrity of injured cardiac tissues. In a combinatorial therapy of SkM and MSCs in infarcted rat increased ejection fraction, neovascularization and muscle fibers in the regions of myocardial fibrosis, indicating reduced ischemia, atrophy, and cardiomyocyte death [103]. When supplemented with fibrin glue as a scaffold, the epicardially implanted SkM prevented wall thinning of the infarcted region and deterioration of LV function in rat [104]. In a long-term clinical study, the surprising presence of engrafted myotubes in post-infarction scar after 16 years of implanted myoblasts indicates its robustness and potential future use in myocardial regeneration [105,106]. Furthermore, to promote the angiogenesis and engraftment of transplanted SkM, the genetic engineering with angiogenic growth factors approach has also been employed. It has been reported that HGF gene-engineered SkM reduced infarct size, fibrosis and collagen deposition, enhanced vessel density, and improved cardiac function in a rat MI model [107]. The modified HGF gene also demonstrated increased myocardial levels of HGF, VEGF, and Bcl-2 and increased the survival and engraftment of SkM. Mechanistically, SkM expressing secretory IL-1 receptor antagonist, a key paracrine mediator of adverse post-MI remodeling exhibited greater graft cell numbers with fewer resultant myotubes in mice MI, suggesting improved cardiac morphology and function through cellular and biochemical changes in components of adverse remodeling. [108]. Besides, the downregulation of apoptosis-regulatory miRNAs led to upregulated levels of target genes, which is attributed to therapeutic effect of SkM in wistar rats [109]. According to a report, both the percutaneous as well as surgical transplantation of autologous myoblasts showed comparable improvements in a mini-pig model of chronic MI, in terms of reversed remodeling and improved systolic function and perfusion, along with decreased scar tissues [110]. Similarly, another study also evidenced an enhanced vasculogenesis and reduced fibrosis in SkM-mediated therapy in swine MI, with no significant difference between groups percutaneous or surgically administered routes [111]. Thus, SkM cells seems promising in MI treatment due to their high proliferative nature and differentiation towards myoblasts. However, the risk of arrhythmia and reduced long-term engraftment limits its potential in developing regenerative therapy for MI [112]. A recent study in porcine model also reported that compared to SkM, the iPSCs mediated MI therapy is much more effective in improving regional contractile function and cardiac bioenergetic efficiency with a better oxygen consumption rate [98].

## 3. Platelet-Derived Biomaterials (PDB)-Based Regenerative Strategies for MI 

PDB are platelet-rich plasma (PRP) releasate, which contains a cargo of growth factors such as PDGF, epidermal growth factor (EGF), TGF-β1, VEGF, FGF, HGF, and IGF-I (Figure 4B), and have been ascribed to their regenerative potential [113,114,115]. Functionally, PRP imparts its regenerative efficacies through neovascularization, angiogenesis, arteriogenesis, and vasculogenesis [113]. In addition, PRP also acts as a scaffold biomaterial for promoting site-specific tissue regeneration [116]. In the MI rat PRP significantly decreased infarct size and increased ventricular wall thickness in the infarcted areas, leading to improved reperfusion, cardiac remodeling and function [117]. In a seminal polytherapy study on Yorkshire pigs, the intramyocardially administered PRP combined with antioxidant and anti-inflammatory molecules decreases LV collagen area fraction and enhanced blood vessel density post-infarction leading to improved ventricular function and attenuated LV remodeling [118]. In a preclinical report, it has also been shown that thrombin-activated PRP could ameliorate LV remodeling through inhibited ventricular expansion and hypertrophy of viable Wistar rat myocardium [119]. This treatment further promoted angiogenesis and arteriogenesis in the infarct regions. In a similar trend, activated PRP through nanosecond pulsed electric fields (nsPRP) enhances LV function in rabbit and rat MI model, suggesting reduced heart irritability and decreased possibility of arrhythmias [120]. nsPRP also increased the expression of heat shock proteins (Hsp) 27 and 70, where Hsp 70 is highly important for suppressing ROS levels to protect vascular endothelium as well as cardiomyocytes from extensive damage. Additionally, nsPRP increased mitochondrial spare respiratory capacity in the presence and absence of H_2_O_2_, providing the heart with increased cardiac capacity, and rendering it less vulnerable to bioenergetic exhaustion during myocardial reperfusion injury. Apart from this, attempts have been made to restore cardiac electrical activity using a nebulizer to minimize surgical or invasive delivery of PRP [121]. The synergistic effect of ADSC embedded in platelet-rich fibrin has also been demonstrated to preserve wall thickness of IA and LV function as well as suppress LV remodeling in a MI rat [122]. This report further implied that ADSCs embedded in PRF scaffold is superior to direct ADSCs implantation for improving cardiac function in a MI. This indicate that PDB released from activated PRP could stimulate proliferation and differentiation of various stem cells in tissue injury. Thus, the PRP possess inexhaustible potential in treatment of MI and other related cardiac disorders. However, limited clinical studies and a lack of standard accepted therapeutic procedures limit the rapid progress in establishing PRP as a therapeutic agent. 

In spite of various significant therapeutic outcomes of regenerative therapies for MI (Table 2), various limitations also exist such as availability of only pre-clinical small animal model such as rodent and rabbits, instead of large animals such as monkeys and pigs to closely mimic the human physiology and validate the pre-clinical efficacy. Further, many pre-clinical MI animal models are immunosuppressed. Hence it is difficult to examine the immune response to transplanted cells. A large amount of these preclinical studies are not based on long-term follow-up, which is required to evaluate the true therapeutic efficacy and possibility of associated adverse events. Even the clinical studies on regenerative therapies are limited to small set of MI patient populations and a lack of standardized procedure reduces their direct use. Other crucial limiting factors for such studies include the availability of regenerative materials to be transplanted, highly-needed expertise for surgical intervention, and expensive treatments. 

## 4. Conclusions

Most stem cells mediate therapeutic function through paracrine activities of their releasate growth factors, which activate a local ischemic microenvironment, rescue cardiomyocytes, promote vasculogenesis, eventually reduce infarct size, and improve LV function. Studies have evidenced that combined or polytherapy of stem cells with hydrogels, nanogels, or PDB is more superior to monotherapy in achieving successful cardiac function. However, a clear picture of the comparative efficacy of various cellular therapies still remains to be elucidated in terms of their doses, time of administration, effect of specific secretomes, and their mechanistic insight. Further, though regenerative therapies may not provide immediate relief in MI, their combination with surgical interventions could be investigated to achieve a high level of performance in recovery.

## Figures and Tables

**Table 1:** Functionalities and signaling pathways of regenerative cellular growth factors. FGF: Fibroblast growth factor, FGFR: Fibroblast growth factor receptor, HGF: hepatocyte growth factor, IGF: Insulin growth factor, MMP: matrix metalloproteinase, PDGF: platelet-derived growth factor, SDF: Stromal-derived factor, TGF: Transforming growth factor, VEGFs: vascular endothelial growth factors.

Growth Factors	Function	Signaling Pathways	Activators/Inhibitors	References
TGF-β (TGF-β1, TGF-β2 and TGF-β3)	Myofibroblast differentiation, ECM protein expression, Macrophage inactivation, down-regulation of cytokine, chemokine and reactive oxygen species, Angiogenic and angiostatic effect, cardiomyocyte hypertrophy	Activation of serine /threonine kinase Smad-mediated regulation of nuclear gene transcription. Activation of Erk, JNK, p38MAPK, Protein Phosphatase 2A (PP2A) and RhoA pathways	Proteases including plasmin, matrix metalloproteinase (MMP)-2 and MMP-9, thrombospondin (TSP)-1, reactive oxygen species and a mildly acidic environment	[8,9,10]
VEGF (VEGF-A, VEGF-B, VEGF-C, VEGF-D)	Promotes vascular endothelial cell growth, survival, and proliferation Also stimulate vasculogenesis, lymphangiogenesis and angiogenesis Myeloid and progenitor cell recruitment, endothelial sprouting	Regulation of angiogenesis through VEGF-A and its interaction with receptor VEGFR-1/Flt-1 VEGFR-2	Anti-VEGF antibody, HIF inhibitors, VEGF-R TK inhibitors (Sorafenib, Sunitinib,	[11,12]
HGF	Anti-apoptotic and anti-autophagic, Angiogenesis, Anti-inflammatory and Immunomodulatory Anti-fibrosis Activation of cardiac stem cells	HGF/Met signaling leading to activation of Intracellular signaling pathways such as Akt/PI3K, Ras (rat sarcoma), MAPK cascades, ERK and Rac1 (Ras-related C3 botulinum toxin substrate 1)	Angiopoietin 1 (Ang1)	[13]
MMPs (MMP-1, MMP-2, MMP-3, MMP-7, MMP-8, MMP-9, MMP-12, MMP-14, MMP-28)	Collagen, fibronectin, proteoglycans and ECM degradation Fragmentation of secreted protein acidic and rich in cysteine (SPARC) resulting in angiogenesis and vascular growth			[14]
SDF-1α	Anti-apoptotic Angiogenesis, homing and mobilization of progenitor cells	SDF-1/CXCR4		[15]
PDGF	Angiogenesis, fibrogenesis, cell proliferation, differentiation, and migration	PDGF/PDGFR mediated phosphatidylinositol 3 kinase, Ras-MAPK, Src family kinases and phospholipase Cγ signaling pathways		[16,17]
IGF-1	Proliferation, gene regulation, autophagy, cell survival and anti-apoptosis	IGF-1/IGF-1R mediated ELK-1,ERKs, PI3K/Akt and mTOR signaling		[18]
FGF	Regulation cardiac remodeling, inhibition of autophagy and control of endoplasmic stress	FGF/FGFR mediated RAS-MAPK, PI3K-AKT and Calcineurin/NEAT signaling		[19]

**Table 2:** Summary of studies on pre-clinical and clinical-based cellular therapies and their main outcomes during treatment of MI. The specific animal used in the pre-clinical studies has been indicated in the bracket ADSCs: Adipose derived stem cells, BMSCs: Bone marrow derived stem cells, CSCs: cardiac stem cells, ESCs: Embryonic stem cells, HGF: Hepatocyte growth factor, IGF-1: Insulin like growth factor-1, iPSC_S_: Induced pluripotent stem cells, LV: Left ventricular, LVEF: Left ventricular ejection fraction, MI: Myocardial infarction, PRF: Platelet-rich fibrin, PRP: Platelet-rich plasma, ROS: Reactive oxygen space, SkM: Skeletal myoblasts.

Cellular Therapy	Pre-Clinical/In Vitro Outcomes	Clinical Outcomes
BMSCs		
BMSC(mice)	Regenerated injured myocardium via BMSC differentiation into myocytes and coronary	
BMSCs (rabbit)	Decreased infarct size	
Shock + autologous BMSC (swine)	Synergistic effect on LVEF, reduced infract size and remodeling	
Exosomes + BMSCs (rat)	Increased neovascularization, reduced infract size	
Tethered IGF-1 + BMSCs (rabbit)	Increased neovascularization, reduced infract size	
BMSCs + Hydrogel	Improvement in systolic and diastolic pressure	
Atorvastatin + MSCs (rat, rabbit and swine model	Improvement in LVEF	
Autologous BMSCs (Clinical)		Reduced infracted size, improved ventricular contraction
Infusion of Bone marrow progenitor cells (Clinical)		Improved regional LV Contractility of infarcted segments, no significant adverse reactions/side effects
Phase II/III clinical trials of autologous bone marrow-derived mononuclear cells		No adverse event and improvement in LVEF
Intracoronary infusion of bone marrow-derived mononuclear cells (BMMC) in ST-segment elevation myocardial infarction (Clinical)		No improvement in infract size, LV function and modeling
ADSCs		
Induction of *in vitro* differentiation of ADSCs using transforming growth factor-β1)	Differentiation of ADSCs into cardiomyogenic cells	
Transfection of ADSCs with T-box 18 gene	Differentiation of ADSCs into cardiomyogenic cells	
Rosuvastatin + ADSCs (mice)	Survival of grafted ADSCs	
Infusion of Ghrelin+ADSCs (mice)	Improved cardiac function, controlled fibrosis and inhibited cellular apoptosis	
ADSCs (rat)	Improved cardiac function and electrophysiological stability	
Primed ADSCs (mice)	Improved LVEF and neovascularization	
Melatonin pretreated ADSCs (rat)	Reduced apoptosis and induced cell proliferation and angiogenesis	
Intravenous infusion of autologous human ADSCs (mice and human)	No adverse effects and tumor formation	No tumor formation and adverse effect
CSCs		
Injection of Adult CSCs in ischemic heart (rat)	Regenerated myocardium and promoted neovascularization	
Infusion of nanogel encapsulated human CSCs in (mice and pig)	Encapsulation protected CSCs from immune attack	
Intracoronary infusion of varying dose of CSCs (rat)	Cellular dose of 6.0 × 10^6^ cells increased the post-operative mortality, improved echocardiographic parameters, reduced apoptosis and infarct size	
Infusion of VEGF and CSCs grafting in poly(l-lactic acid) mat (rat)	Additive effect on improvement in angiogenesis and cardiomyogenesis *in vitro* and animal	
Infusion of synthetic cell-mimicking microparticles rich in proteins and membranes of CSCs (mice)	Improved cardiac functional without any adverse immune response	
Infusion of allogenic human CSCs in MI patients clinical I/II study		Improvement in LVEF without any significant adverse reaction and immune response
ESCs		
Transplantation of human ESCs and derived cardiomyocytes (rat)	ESCs formed teratoma-like structures and functional loss. Whereas ESCs derived cardiomyocytes showed structural functional and recovery *in vivo*	
Transplantation of committed mouse ESCs (sheep)	Significantly improved LVEF	
ESCs derived cardiac progenitor cells in treatment of heart failure (human)		Functional and contractile improvement
ESC derived exosomes in (mice)	Cardiac regeneration without teratoma formation	
Transplantation of Human ESCs derived cardiomyocytes (macaques)	Improved LVEF with abnormal electrical pulse	
iPSCs		
Infusion of iPSCs-derived progenitor cells (mouse)	Improved cardiac function, differentiation towards cardiac lineage	
Infusion of non-human and human iPSCs-derived cardiomyocytes (rodent)	Improved cardiac function	
Infusion of iPSCs-derived cardiomyocytes encapsulated polyethylene glycol hydrogel (rat)	Inhibited ventricular remodeling, enhanced ejection fractions	
Transplantation of engineered human heart tissue derived from ADSCs (guinea pig)	Improved re-muscularization of infracted area	
Infusion of cardiac muscle patch in fibrin developed from iPSCs derived cardiomyocytes (swine)	Improved LVEF and cardiac function Reduction infract size and LV wall stress	
Human iPSCs derived cardiomyocytes (rat)	Improved cardiac function and electrical conduction	
Myoblast sheet transplantation (rat)	Effective in the infant MI rat heart compared to young one	
Transplantation of SkM+ mesenchymal cells (rat)	Neovascularization and muscle fiber formation	
Long term engrafting of myoblast in infarcted heart (human)		Long-term LVEF improvement and survival of myoblasts
Over-expression of HGF in SkM (rat)	Control in infarct size and collagen deposition	
PRP		
Autologus PRP under oxidative stress (rabbit)	Improvement in LVEF and reduction in scar size	
Injection of PRP in myocardium (Fisher rat)	Improvement in LVEF and reduction in ROS formation	
Autologous PRP (pig)	Improved ejection fraction and myocardial reperfusion	
Injection of thrombin activated PRP (rat)	Structural and functional recovery, controlled LV remodeling	
Infusion of nano second electric pulse activated PRP (rabbit and mouse)	Improved LVEF and myocardial reperfusion	
Transplantation of PRF embedded ADSCs (rat) model	Improved LV function and controlled LV remodeling	
